# Re-recruiting postpartum women living with HIV into a follow-up study in Cape Town, South Africa

**DOI:** 10.1186/s13104-019-4509-4

**Published:** 2019-07-26

**Authors:** Phepo Mogoba, Yolanda Gomba, Kirsty Brittain, Tamsin K. Phillips, Allison Zerbe, Landon Myer, Elaine J. Abrams

**Affiliations:** 1 Division of Epidemiology and Biostatistics, School of Public Health and Family Medicine, University of Cape Town, Cape Town, South Africa; 2 Centre for Infectious Disease Epidemiology and Research, School of Public Health and Family Medicine, University of Cape Town, Level 5, Falmouth Building, Anzio Road, Cape Town, South Africa; 3 ICAP at Columbia University, Mailman School of Public Health, Columbia University, New York, USA; 4 Department of Pediatrics, Vagelos College of Physicians and Surgeons, Columbia University, New York, USA

**Keywords:** Recruitment, Retention, Postpartum, HIV, South Africa

## Abstract

**Objective:**

Recruitment and retention present major challenges to longitudinal research in maternal and child health, yet there are few insights into optimal strategies that can be employed in low-resource settings. Following prior participation in a longitudinal study following women living with HIV through pregnancy and breastfeeding in Cape Town, women were re-contacted at least 18 months after the last study contact and were invited to attend an additional follow-up visit. We describe lessons learnt and offer recommendations for a multiphase recruitment approach.

**Results:**

Using telephone calls, home visits, clinic tracing and Facebook/WhatsApp messages, we located 387 of the 463 eligible women and successfully enrolled 353 (91% of those contacted). Phone calls were the most successful strategy, yielding 67% of enrolments. Over half of the women had changed their contact information since participation in the previous study. We recommend that researchers collect multiple contact details and use several recruitment strategies in parallel from the start of a study. Participants in longitudinal studies may require frequent contact to update contact information, particularly in settings where mobility is common.

**Electronic supplementary material:**

The online version of this article (10.1186/s13104-019-4509-4) contains supplementary material, which is available to authorized users.

## Introduction

Recruitment and retention, crucial to the success of research studies, are hampered by numerous challenges in low- and middle-income countries. Withdrawal and loss from study follow-up is a particular concern in longitudinal studies [1, 2]. Poverty-related factors including informal housing, mobility, changing contact details and competing priorities such as child care and employment, present barriers to continued study participation and participant tracking [3, 4]. For HIV-related research specifically, HIV-related stigma and risks related to inadvertent HIV-status disclosure are barriers to recruitment and retention [3].

Traditional recruitment methods include health facility-based recruitment, snowball sampling, using index participants or invitation letters, and recruitment from other study populations or from existing databases [1, 2, 5]. Common tracking methods include telephone follow-up, home visits and contacting pre-identified contact persons [6]. Additionally, the rapid growth of digital technology has led to use of social media for recruitment and follow-up, however the feasibility and success of these methods in low-resource settings have not been reported [7–9]. We present lessons learnt from recruiting women living with HIV (WLHIV) into a follow-up study following prior participation in a longitudinal study of health service delivery approaches during pregnancy and the postpartum period in South Africa.

## Main text

### Methods

#### Setting

The MCH-ART study (Maternal Child Health Antiretroviral Therapy, ClinicalTrials.gov NCT01933477), a multi-phase implementation science study evaluating strategies for delivering HIV care to pregnant and postpartum women, was conducted in the former township of Gugulethu in Cape Town. Gugulethu is a predominantly Black/African community with high levels of unemployment and poverty [10], and migration into and out of the community from neighbouring communities and other provinces [11].

#### MCH-ART study

In MCH-ART, 471 breastfeeding women who had initiated antiretroviral therapy (ART) during pregnancy (April 2013–June 2014) were enrolled into a postpartum trial and followed through 18 months postpartum. Women were randomised to receive either integrated postnatal care and ART services within the maternal and child health setting where they had received antenatal care (the MCH intervention), or the local standard of care (SOC). The primary aim of the study was to compare maternal viral suppression and retention across services. The study design has been published previously [12, 13]. Women provided detailed locator information, including personal telephone numbers, home addresses, and telephone numbers of friends/family that they did and did not live with. All women consented to study staff contacting them using either their own numbers/addresses or family/friend’s phone numbers for future research.

#### LACE study

The LACE (long term adherence and care engagement) study assessed health outcomes among women enrolled in the MCH-ART trial at a single study visit at 36–60 months postpartum. This visit occurred at least 18 months after the last MCH-ART study contact. Excluding those who had withdrawn (n = 5) or died (n = 3), 463 participants were eligible to attend the LACE study visit.

#### Analysis

Data analyses were conducted using Stata (Stata Corporation, College Station, Texas). The recruitment strategies undertaken for the LACE study were described across months of recruitment. The final recruitment outcome (enrolled, refused participation, relocated, deceased, located but did not attend) and last attempted strategy for all located women were enumerated. Women who were successfully enrolled and those not enrolled were compared using Chi square, rank sum or Kruskal–Wallis tests as appropriate. Among women who were enrolled, the time from date of the first contact attempt to enrolment was compared across socio-demographic characteristics.

### Description of recruitment strategies

**Fig. 1:**
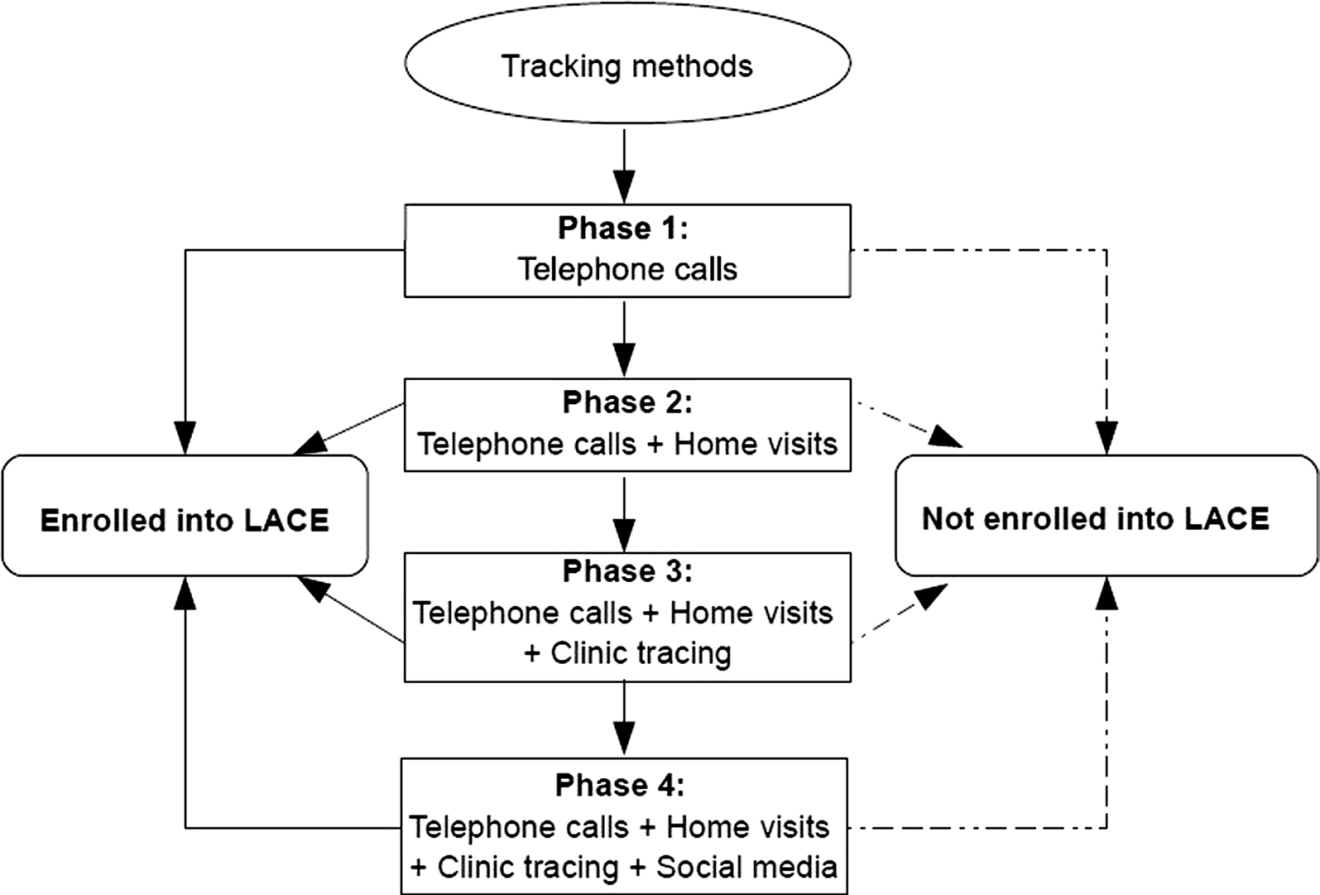
The recruitment process followed for each individual participant in the LACE study

Recruitment was conducted from April 2017 to March 2018. The research team, including experienced recruitment staff, attended workshops prior to and 6 months into the study to brainstorm recruitment strategies. Upon identification of appropriate strategies, we divided our recruitment process into four phases summarized in Fig. 1. The order of strategies was informed by the level of effort required, i.e. we started with the least resource-intensive strategy (telephone calls) before moving on to more resource-intensive strategies. To ensure we maintained participant confidentiality across all recruitment strategies, once contact was made we used scripted messages that made no reference to HIV specifically to establish if we were speaking to the correct woman (Additional file 1: Table S1). Following a successful contact, an appointment for a study visit was made.

#### Phase 1: Telephone calls

Recruitment began with telephone calls. Participants were contacted for two consecutive days during business hours and if unsuccessful, two additional attempts were made outside of business hours. Continued telephonic contact attempts, at least monthly, were made throughout the recruitment period, while simultaneously using the additional strategies.

#### Phase 2: Phase 1 strategy and home visits

Following unsuccessful telephonic contact, the team conducted home visits during weekdays and weekends via car with a driver who operated a local taxi service. Given challenges in locating homes in informal settlements, we solicited the help of local community representatives or “street committees” to assist. Like telephonic contact, continued home visits were conducted even when other recruitment methods were employed. A home visit was rarely successful on the first attempt. The recruitment team visited the same address multiple times before locating the participant or concluding that they no longer lived there if, for example, the current occupant(s) provided a new address.

#### Phase 3: Phase 2 strategies and routine clinic contact

If initial telephonic contacts and home visits proved unsuccessful, we searched routinely-collected electronic medical data using participant’s name and date of birth to ascertain where she was receiving HIV care, and the date of her next appointment. Permission to recruit from these clinics was obtained from the Provincial Department of Health and local facility managers. On the day of the scheduled appointment, our recruiters attempted to locate the participant at the clinic or placed an invitation letter in her clinic folder.

#### Phase 4: Phase 3 strategies, Facebook and WhatsApp

Facebook and WhatsApp were added as recruitment strategies 6 months into the recruitment period. Using a Facebook account created for the study, we searched for women by name and surname, region and date of birth if these details were made public on their Facebook profile. Once we were reasonably sure that a Facebook account belonged to a participant, we sent one standardized private message that included the study contact details. It explained that the recipient could ignore the message if they were not interested or if they were not the person we were looking for (Additional file 1: Table S1).

WhatsApp is a popular messaging platform in this setting, but it is common for people to use different cellphone numbers for WhatsApp and phone calls. We attempted to contact women via WhatsApp using the cellphone number provided during the MCH-ART study even if we were unable to contact them telephonically using the same number. Our recruiters started conversations on WhatsApp using language described in Additional file 1: Table S1. If a participant was contacted, we would ask for her current phone number for voice calls and call her for recruitment.

#### Participant-driven recruitment

Participants who were successfully recruited were asked if they were familiar with other women who had participated in the MCH-ART study and received a R160 (USD ~ 11) voucher incentive if they referred another woman to the study. In addition, a few women who we had not established direct contact with walked in after being informed by a family member/friend who we had reached.

### Results of recruitment attempts

**Fig. 2:**
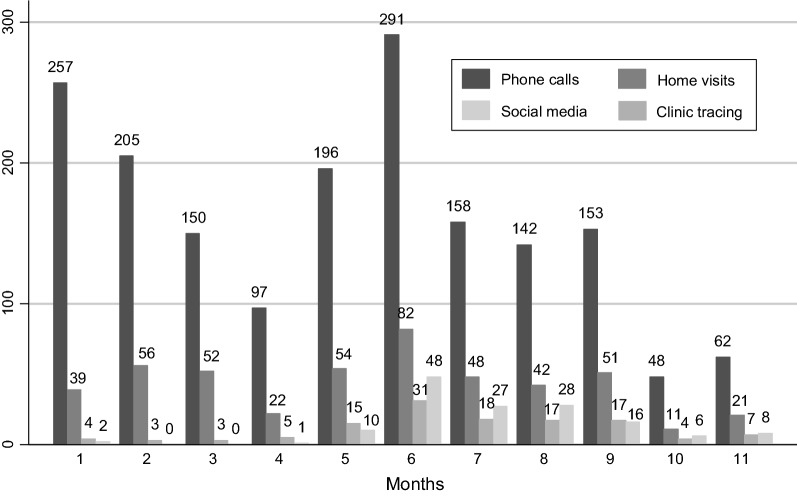
Total number of recruitment attempts by month and recruitment strategy

Of 463 women eligible for the LACE study, 387 (84%) were contacted and 353 (76%) enrolled. Overall, the recruitment team made 1759 phone calls, 478 home visits, 124 clinic tracing attempts, and 47 Facebook/WhatsApp contact attempts (Fig. 2) to successfully contact the 387 women. Of these, we successfully recruited 353 (91%) through a total of 1306 phone calls (median: 3; range 1–17) and 336 home visits (median: 1; range 1–5). Table 1 presents the final recruitment strategies for women who consented to participate in the LACE study: the majority were ultimately successfully recruited using phone calls (67%); with a smaller proportion recruited through home visits (29%); and a minority (4%) using personal referrals, clinic tracing and Facebook/WhatsApp.

**Table 1:** Recruitment methods and outcomes of the last contact attempt for located women (n = 387)

	Enrolled	Not enrolled in LACE				Total number of women located
		Refused participation	Relocated	Deceased^a^	Located but did not attend	
Number of women	353 (91)	6 (1)	3 (1)	7 (2)	18 (5)	387 (100)
Phone calls	238 (67)	3 (50)	1 (33)	4 (57)	16 (89)	262 (68)
Home visits	101 (29)	1 (17)	2 (67)	3 (43)	2 (11)	109 (28)
Clinic tracking	2 (1)	0	0	0	0	2 (< 1)
Social media	5 (1)	0	0	0	0	5 (1)
Referral^b^	2 (1)	0	0	0	0	2 (< 1)
Walk-in^c^	5 (1)	2 (33)	0	0	0	7 (2)

^a^Participants reported as deceased

^b^Participants referred to study by enrolled participants

^c^Participants who came for a visit after being informed by alternative contact we had previously reached

Women who were contacted sooner after their last MCH-ART visit were more likely to be successfully reached and recruited (Additional file 1: Tables S2, S3). Of women enrolled, over 50% consented within 2 months of the first contact attempt. Compared to women enrolled in later months of the study, those who were enrolled earlier had higher levels of education and were more likely to be engaged in HIV care. Further, women who were contacted closer to their last MCH-ART visit were less likely to have changed their original contact details (p = 0.002; Additional file 1: Table S3). Among all women enrolled, 209 (59%) had changed their locator details since participation in the MCH-ART study: 55% changed their phone number, 20% changed their residential address, and 25% changed both phone number and address. We found that 32 women had relocated outside of the Western Cape; 27 agreed to return for the study visit.

After a total of 453 phone calls (median: 7; range 4–13) and 142 home visits (median: 3; range 2–7), among those not enrolled in LACE, 76 women were never successfully contacted. There were few appreciable differences between women who were enrolled and those not enrolled (Additional file 1: Table S2).

### Discussion

Using both traditional and novel recruitment strategies, 76% of women who had previously participated in the MCH-ART trial were successfully re-recruited for the LACE study at least 18 months after the last visit in MCH-ART. In sharing these experiences, we intend to describe strategies that may assist research teams doing similar work.

In line with other research among WLHIV in South Africa, we observed high levels of residential instability and mobility [11, 14, 15]. Maintaining frequent communication has been noted as a critical retention strategy [16]; here, population mobility affected the ease of locating participants after 18 months without updated contact details. Researchers should carefully consider the frequency of eliciting changes in contact information and what information to collect, including addresses of relatives and rural homes In addition, researchers should assess participant preferences related to the use of contact details and implement strategies to ensure confidentiality is maintained during any communication process. These strategies may make it easier to locate participants in the long term and lower the intensity and costs of contact attempts [16]. Researchers should also formulate logistical and financial plans for recruiting and retaining participants who have relocated to other areas.

Previous studies have suggested that different communication strategies affect study completion rates [17, 18]. Here, phone calls were the most successful strategy, possibly due to continued telephonic contact attempts throughout the recruitment period. Additionally, our study supports previous findings that collecting details of more alternate contacts significantly improves study retention [19]. Given improvements in mobile phone coverage and wireless infrastructure in developing countries, researchers should consider collecting email addresses, social media and similar contact details that are not affected by changes in mobile phones or residential address [20, 21].

Finally, we believe that a critical factor in the success of our multiphase approach was the flexibility and creativity of our recruitment team. The team members resided in the study area and had worked on other studies in this setting. We suggest that researchers pay as much attention to the composition of and continued engagement with their recruitment team as they do to their recruitment methods.

### Conclusions

Our findings show that a multiphase, parallel recruitment approach can successfully reach a large proportion of study participants. High turnover of contact information was the biggest barrier to recruitment. Based on these findings, we recommend that researchers in similar settings employ multiple strategies simultaneously to re-recruit or retain study participants. Strategies must be appropriate for the setting and participants being traced, and the use of digital methods should be increasingly considered as populations increase their use of these platforms.

## Limitations

The use of Facebook and WhatsApp was not as successful as anticipated, possibly due to initiating this strategy later in the recruitment period, and thus contacting women who were more difficult to locate. In future, researchers should consider using all possible recruitment methods from the beginning of a study. Unfortunately, participant perceptions of each recruitment approach were not formally assessed. Further research on this could help to tailor recruitment strategies. Although the focus of our work was on re-recruitment of an existing cohort at one study site, we believe that the same communication strategies may be applicable in similar populations and for retaining participants in long-term cohort studies.

## Additional file

**Additional file 1.** Additional tables.

## Data Availability

Data will be made available to readers upon a reasonable request to the corresponding author.

## Abbreviations

WLHIV: women living with HIV
MCH-ART: Maternal Child Health Antiretroviral Therapy
ART: antiretroviral therapy
SOC: standard of care
LACE: long term adherence and care engagement
