# Supplementary material for: Re-recruiting postpartum women living with HIV into a follow-up study in Cape Town, South Africa
Source: BMC Res Notes. 2019 Jul 26;12:461. doi: 10.1186/s13104-019-4509-4 (PMC6660934; doi:10.1186/s13104-019-4509-4)
Supplement: Supplementary file 1 — Additional file 1. Additional tables. [file 13104_2019_4509_MOESM1_ESM.docx]

**Additional material**

**Additional file 1: *Table S1:*** *Recruitment Script Guidelines and language*

| **Recruitment script guidelines for telephone calls and home visits** |
| --- |
| - Ask to speak to the participant - Introduce yourself by stating your name and where you are calling from [insert clinic name] - Confirm that you are speaking to participant before talking about study details - Tell participant name of study you are calling from - Tell them that you called them because they previously participated in MCH-ART and agreed to be contacted for future studies - Describe the LACE study: one visit, questionnaires, blood drawn only from mother, and should come with the child - Describe reimbursement: we will thank you by giving you a R150 (USD~10) Shoprite voucher, a gift for the child and reimburse your transport costs. This is not payment for participating but is reimbursement for your time. |
| **Language used in the clinic invitation letter, Facebook and WhatsApp recruitment** |
| “Dear [insert name], we are from the University of Cape Town and we are trying to  follow up with mothers who received antenatal care at the [insert clinic name]  in 2013 and 2014. We’d like to find out how you and your children are doing now,  after 3-4 years.  If you were attending the [insert clinic name] for antenatal  care during that time, and would be interested in taking part in our follow-up, please  contact us for more details. You can reply to this message or send a message or  “please call me” to [insert study phone number].  If you are not interested in being part of the follow-up you can either message us to  let us know or you can simply ignore this message.  If you did not attend the [insert clinic name] in 2013/2014 and we have contacted you in  error, we apologise for this, and you can ignore this message.  We look forward to hearing from you.” |

**Additional file 1: *Table S2:*** *Characteristics of women eligible for participation in LACE compared by enrolment status*

| **Characteristic** | **Total women eligible for LACE** | **Enrolled in LACE** | **Not enrolled in LACE** | **Difference between not enrolled and enrolled** |
| --- | --- | --- | --- | --- |
| Number of women | 463 (100) | 353 (76) | 110 (24) | p value |
| Mean age (SD) | 28.5 (5.4) | 28.6 (5.4) | 28.1 (5.5) | 0.324 |
| Home language: isiXhosa | 448 (97) | 345 (98) | 103 (94) | 0.034 |
| Median highest level of education | 11 [10,110 | 11 [10,11] | 11 [10, 12] | 0.371 |
| Educational attainment: Less than secondary | 348 (75) | 267 (76) | 81 (74) |  |
| Completed secondary/any tertiary | 115 (25) | 86 (24) | 29 (26) | 0.671 |
| Currently employed | 180 (39) | 141 (40) | 39 (35) | 0.399 |
| Housing type: Informal | 257 (56) | 192 (54) | 65 (60) |  |
| Formal | 205 (44) | 161 (46) | 44 (40) | 0.336 |
| Married/cohabiting | 190 (41) | 136 (39) | 54 (49) | 0.049 |
| Multiparous | 377 (81) | 294 (83) | 83 (75) | 0.065 |
| Median gestation age at first ANC visit (IQR) | 21 [16, 27] | 21 [16, 26] | 21 [17, 27] | 0.485 |
| Timing of first ANC |  |  |  |  |
| First trimester | 79 (17) | 61 (17) | 18 (16) |  |
| Second trimester | 276 (60) | 211 (60) | 65 (59) |  |
| Third trimester | 108 (23) | 81 (23) | 27 (25) | 0.932 |
| Newly diagnosed HIV+ in this pregnancy | 263 (57) | 194 (55) | 69 (63) | 0.151 |
| Median months between last MCH-ART visit and first contact in LACE (IQR) | 25 [23,31] | 25 [23,31] | 29 [23,33] | 0.003 |

**Additional file 1: *Table S3:*** *Characteristics of women enrolled in LACE compared by months between the first contact and enrolment*

| **Characteristic** | **Total number of women enrolled in LACE** | **<2 months** | **≥2 to <6 months** | **≥6 months** | **Differences between women** |
| --- | --- | --- | --- | --- | --- |
| Number of women | 353 (100) | 193 (55) | 122 (34) | 38 (11) | p value |
| ***Characteristics at MCH-ART visit*** |  |  |  |  |  |
| Mean age (SD) | 28.6 (5.4) | 29.2 (5.7) | 27.8 (5.1) | 28.5 (4.8) | 0.152 |
| Median highest level of education | 11 [10,11] | 11 [10, 12] | 11 [10, 11] | 11 [10, 11] | 0.015 |
| Completed secondary/any tertiary | 86 (24) | 60 (31) | 23 (19) | 3 (8) | 0.002 |
| Employed | 141 (40) | 87 (45) | 41 (34) | 13 (34) | 0.096 |
| Housing type: Informal | 181 (51) | 97 (50) | 61 (50) | 23 (61) |  |
| Formal | 172 (49) | 96 (50) | 61 (50) | 15 (39) | 0.482 |
| Married/cohabiting | 136 (39) | 77 (40) | 45 (37) | 14 (37) | 0.845 |
| Multiparous | 294 (83) | 162 (84) | 102 (16) | 30 (21) | 0.748 |
| Newly diagnosed HIV+ in this pregnancy | 194 (55) | 107 (55) | 65 (53) | 22 (58) | 0.865 |
| ***Characteristics at LACE visit*** |  |  |  |  |  |
| Mean age (SD) | 32.6 (5.4) | 33 (5.7) | 31.9 (5.1) | 33 (4.8) | 0.210 |
| Median Parity (IQR) | 2 [2, 3] | 2 [2, 3] | 2 [2, 3] | 2.5 [2, 3] | 0.917 |
| Completed secondary/any tertiary education | 110 (31) | 69 (36) | 34 (28) | 7 (18) | 0.068 |
| Currently employed | 170 (48) | 95 (49) | 57 (47) | 18 (47) | 0.906 |
| Married /cohabitating | 134 (38) | 77 (40) | 46 (38) | 11 (29) | 0.445 |
| Had a repeat pregnancy after MCH-ART | 68 (19) | 32 (17) | 27 (22) | 9 (24) | 0.365 |
| Median months between last MCH-ART visit and first contact in LACE (IQR) | 25 [23, 31] | 25 [22, 26] | 25 [23, 31] | 31 [26, 33] | <0.001 |
| Engaged in HIV care | 238 (67) | 143 (74) | 74 (61) | 21 (55) | 0.011 |
| Changed locator details | 209 (59) | 99 (51) | 81 (66) | 29 (76) | 0.002 |
| Changed phone number only | 115 (55) | 52 (53) | 46 (55) | 17 (58) |  |
| Changed home address only | 41 (20) | 24 (24) | 10 (12) | 7 (24) |  |
| Changed both phone number and home address | 53 (25) | 23 (23) | 25 (31) | 5 (17) | 0.005 |
| Median phone calls (min, max), n=347 | 3 [1, 14] | 2 [1, 5] | 3 [1, 13] | 6 [1,14] | <0.001 |
| Median home visits (min, max), n=186 | 1 [1,5] | 1 [1, 3] | 1 [1,5] | 2 [1, 5] | <0.001 |
